# Transcriptomic Prediction of Pig *Liver-Enriched Gene 1* Functions in a Liver Cell Line

**DOI:** 10.3390/genes11040412

**Published:** 2020-04-10

**Authors:** Zhe Zhang, Zizengchen Wang, Yanna Dang, Jinyang Wang, Sakthidasan Jayaprakash, Huanan Wang, Jin He

**Affiliations:** 1 Department of Animal Science, College of Animal Sciences, Zhejiang University, Hangzhou 310058, China; zhe_zhang@zju.edu.cn (Z.Z.); 11717011@zju.edu.cn (Y.D.); wangjinyang@zju.edu.cn (J.W.); 2 Department of Veterinary Medicine, College of Animal Sciences, Zhejiang University, Hangzhou 310058, China; 21817097@zju.edu.cn (Z.W.); hnwang@zju.edu.cn (H.W.); 3 Department of Chemical Engineering, Hindustan Institute of Technology and Science, Chennai 603103, India; jsdasan@hindustanuniv.ac.in

**Keywords:** *LEG1*, RNA-seq, functional prediction, pig

## Abstract

The newly identified liver-enriched gene 1 (*LEG1*) encodes a protein with a characteristic domain of unknown function 781 (DUF781/LEG1), constituting a protein family with only one member in mammals. A functional study in zebrafish suggested that *LEG1* genes are involved in liver development, while the platypus *LEG1* homolog, Monotreme Lactation Protein (*MLP*), which is enriched in the mammary gland and milk, acts as an antibacterial substance. However, no functional studies on eutherian *LEG1s* have been published to date. Thus, we here report the first functional prediction study at the cellular level. As previously reported, eutherian *LEG1s* can be classified into three paralogous groups. Pigs have all three *LEG1* genes (*pLEG1s*), while humans and mice have retained only *LEG1a*. Hence, *pLEG1s* might represent an ideal model for studying *LEG1* gene functions. RNA-seq was performed by the overexpression of *pLEG1s* and platypus *MLP* in HepG2 cells. Enrichment analysis showed that *pLEG1a* and *pLEG1b* might exhibit little function in liver cells; however, *pLEG1c* is probably involved in the endoplasmic reticulum (ER) stress response and protein folding. Additionally, gene set enrichment analysis revealed that platypus *MLP* shows antibacterial activity, confirming the functional study in platypus. Therefore, our study showed from the transcriptomic perspective that mammalian *LEG1s* have different functions in liver cells due to the subfunctionalization of paralogous genes.

## 1. Introduction

Limited information is currently available concerning the biological functions of liver-enriched gene 1 (*LEG1* or *C6orf58* homolog), which encodes a protein with the characteristic domain of unknown function 781 (DUF781 or LEG1 domain). The name of the gene is based on its enrichment in zebrafish liver, and it was experimentally shown to participate in liver development and to mediate an anti-stress pathway during liver development [1,2]. Two *LEG1* copies are found in the zebrafish genome (*zleg1a* and *zleg1b*), which exhibit functional redundancy. The encoded LEG1 proteins are glycosylated secreted proteins, as indicated by several potential N-glycosylated sites and a signal peptide preceding the LEG1 domain. These features have been validated in numerous proteomic studies in mammals, in which glycosylated LEG1 proteins are present in the saliva, milk, and seminal plasma [3,4,5,6,7]. However, the only functional study of mammalian *LEG1* thus far was carried out in platypus, in which the mammary gland- and milk-enriched *LEG1* homolog Monotreme Lactation Protein (*MLP*) plays a role in antibacterial activity during the nipple-less delivery of milk to pups [8]. To elucidate the function of *LEG1* genes, we previously conducted a phylogenetic analysis using all the available LEG1 protein information, which was predicted based on the presence of the LEG1 domain [9]. In that study, mammalian *LEG1* genes could be classified into three separate clades: *LEG1a*, *LEG1b*, and *LEG1c*. These different clades might have undergone subfunctionalization, thus conferring different functions upon each clade. Among the species included in the analysis, humans and mice only have one copy of the *LEG1* gene, belonging to *LEG1a* (*hLEG1a* and *mLeg1a*). Pigs, on the other hand, exhibit all three copies, designated *pLEG1a*, *pLEG1b*, and *pLEG1c*, the last of which phylogenetically clusters with platypus *MLP*. Therefore, platypus *MLP* and *pLEG1c* might share similar functions, while the orthologous genes *hLEG1a*, *mLeg1a*, and *pLEG1a* preserve other functions [9].

To decipher the biological roles of each clade of mammalian *LEG1* genes, we performed a gain-of-function study. The three pig *LEG1* genes were overexpressed in the HepG2 liver cell line to determine whether the overexpression of *LEG1* genes could result in biological alterations in the cell line. We first predicted the functions of the genes using two bioinformatics tools. Then, RNA-seq and enrichment analyses were employed to investigate the results. We show here that *pLEG1a* and *pLEG1b* play few roles in hepatic cells, while *pLEG1c* might be involved in misfolded protein processing and endoplasmic reticulum stress responses. We also confirmed that some antibacterial Gene Ontology (GO)/Kyoto Encyclopedia of Genes and Genomes (KEGG) terms were altered by overexpressing platypus *MLP*. Therefore, the different clades of *LEG1* genes might have experienced subfunctionalization after duplication in mammalian genomes.

## 2. Materials and Methods

### 2.1. Cell Culture and Transfection

HepG2 cells were purchased from the American Type Culture Collection (ATCC, Manassas, VA, USA) and cultured in Dulbecco’s Modified Eagle Medium (HyClone, GE, Beijing, China) with 10% fetal bovine serum (HyClone, GE, Beijing, China). Transfection reactions were performed when 1 × 10^6^ cells reached 80% confluency in 6-well plates with 3 micrograms of endotoxin-free plasmids (Endofree Mini Plasmid Kit II, Tiangen, Beijing, China) using Lipofectamine 3000 (Thermo Fisher, Shanghai, China) according to the manufacturers’ guidelines. For each plasmid, transfections were carried out at least three times.

### 2.2. Plasmid Preparation

The pig *LEG1* gene expression plasmids pCAG-pleg1a-3×FLAG, pCAG-pleg1b-3×HA, and pCAG-pleg1c-3×FLAG were constructed previously [9]. Using similar strategies, the pCAG-platypusMLP-3×FLAG plasmid was generated. Briefly, the platypus *MLP* coding sequence (NM_001323776) was modified by adding a Kozak site to the 5′ end and a 3×FLAG sequence to the 3′ end. Then, two *Not*I sites were added to either end. Thereafter, the designed sequence (*Not*I-Kozak-*MLP*-3×FLAG-*Not*I) was synthesized by GenScript (Nanjing, China) and subcloned into the pUC57 vector. Finally, the *Not*I-digested fragment from the pUC57 vector was gel purified (Thermo Fisher, Shanghai, China) and subcloned into the *Not*I site of the pCAG-floxP-neo plasmid to generate the pCAG-platypusMLP-3×FLAG plasmid. The empty vector (pCAG-floxP-neo) without the *LEG1* gene was used as a control. The schemes of four *LEG1* containing plasmids are shown in Figure S1.

### 2.3. RNA Extraction, Reverse-Transcription PCR (RT-PCR), Quantitative Real-Time PCR (qRT-PCR), and RNA-seq

One million nontransfected HepG2 cells were first subjected to RNA extraction using a Total RNA Kit I (Omegabiotek, Guangzhou, China). First-strand cDNA was synthesized using the M-MLV kit from Promega (Madison, WI, USA). RT-PCR was carried out in a 15 μL reaction containing either *hLEG1a*-specific primers (hLeg1a-F: TTTATGGGGGTTGCCTCTGC, hLeg1a-R: CCACAGTTTGTCCTTCGGGT) or human *ACTB* primers (hbactin-F: CCCCAAGGCCAACCGCGAGAAGAT, hbactin-R: AGGTCCCGGCCAGCCAGGTCCAG).

Forty-eight hours after the transfection of HepG2 cells with different plasmids, total RNA was prepared using the MiniBEST Universal RNA Extraction Kit (Takara, Dalian, China). One microgram RNA was used for subsequent qRT-PCR validations, which were performed in a 15 μL reaction with 7.5 μL SYBR Green (Takara, Dalian, China), 0.2 μM primer, and 1 μL reversely transcribed cDNA. The primers for qRT-PCR are as follows: *pLEG1a* (pLeg1a-F: GGATGCACACATTTCAGCCT, pLeg1a-R: ACAAGGACTCGTGGTGGTAG), *pLEG1b* (pLeg1b-F: CCCTCTTCCCTACGACTTTGA, pLeg1b-R: AACAAGCATTCGTGTTGGC), *pLEG1c* (pLeg1c-F: GCATCGGCTTCTATGTGCTT, pLeg1c-R: TGCCTCTATCCATAGCTCGTG), platypus *MLP* (MLP-F: TCATTCCCTTTCAGGTGGCT, MLP-R: AAGTGGAGACTGAGCTGGAC), and human *ACTB* (hbactin-F and hbactin-R).

Then, the rest of the RNA was used for library construction and subjected to sequencing on the Illumina HiSeq X Ten platform (San Diego, CA, USA) and quality control was according to the pipeline of Novogene (Beijing, China). The software Salmon [10], which can efficiently quantify transcript abundance, without aligning reads to a reference genome, was used to quantify transcription levels with a human target transcriptome as a reference. The target transcriptome used here was composed of human genes’ cDNA sequences and the plasmid sequences containing pig *LEG1* genes and platypus *MLP* gene. After expression profiles of these genes were measured, differentially expressed gene (DEG) set (adjusted *p*-value < 0.05 and |log_2_fold change| > 0.5849) identification was then performed using DESeq2 [11]. Sample-level principal component analysis (PCA) was conducted based on the variance stabilization transformation of counts (*vst* function of DESeq2). The RNA-seq data were submitted to the Gene Expression Omnibus under accession number GSE145329.

### 2.4. Enrichment Analysis

The DEGs were first subjected to searches with either the Database for Annotation, Visualization and Integrated Discovery (DAVID) tool [12,13] or the Molecular Signatures Database (MSigD) [14] to identify significantly enriched Gene Ontology (GO) [15] terms and Kyoto Encyclopedia of Genes and Genomes (KEGG) [16] pathways using default parameters (EASE score/modified Fisher’s exact *p*-value < 0.1 or false discovery rate (FDR) < 0.05, respectively).

Then, gene set enrichment analysis (GSEA) [14] was performed for each gene set using GSEA (v.4.0.3, Broad Institute Inc., San Diego, CA, USA) with default parameters (1000× permutation; Signal2Noise as the metric for ranking genes). C5 collection (GO) and C2 (KEGG) were utilized to identify GO terms and KEGG pathways that were differentially regulated in different comparisons. Significantly enriched terms were identified according to a *p*-value < 0.05, FDR < 0.25, and |Normalized Enrichment Score (NES)| > 1.

### 2.5. Bioinformatic Prediction of the Functions of LEG1 Proteins

Two bioinformatics tools were employed: PANDA and I-TASSER, which predict protein functions based on the homolog searches and protein structure, respectively [17,18]. Default parameters were used by submitting the following sequences: pLEG1a (XP_003121259.1), pLEG1b (XP_020930551.1), pLEG1c (XP_020940144.1), and platypus MLP (NP_001310705.1).

### 2.6. Statistics

One-way ANOVA with Tukey’s honestly significance difference (HSD) post hoc method was used to compare the expression levels (transcripts per million, TPM) of the *hLEG1a*, *pLEG1a*, *pLEG1b*, *pLEG1c*, and platypus *MLP* genes in different transfection groups. Two-way ANOVA with interaction effect (group × gene) combined with Tukey’s HSD were then employed to measure the exogenous gene expression levels measured by the qRT-PCR in each group. Data are presented as the means ± standard errors of the mean (SEMs). Type I error was set at 0.05.

### 2.7. Ethical Statement

The current experiment was conducted according to the guidelines of China Council on Animal Care and Protocol and approved by the Animal Welfare Committee of Zhejiang University (no. 11834 issued on 26 February 2018).

## 3. Results

### 3.1. Bioinformatic Prediction of LEG1 Functions

Currently, insufficient information regarding the function of LEG1 proteins is available. Thus, in our study, in silico methods were first used to predict the functions of these proteins. PANDA predicted that pLEG1a, pLEG1b, pLEG1c, and platypus MLP are all secreted proteins involved in multicellular organism development, while pLEG1c and MLP play additional roles in the liver and digestive system development (Table S1). In contrast, the I-TASSER method showed that these LEG1 proteins were all involved in respiration-related processes and neural cell-related processes (Table S2). The cellular components predicted by I-TASSER belonged to the intracellular, plasma membrane, and mitochondrial inner membrane categories, in contrast to the results of PANDA (Tables S1 and S2). Overall, the *in silico* predictions produced by the two approaches raised fairly different results, necessitating the experimental prediction method.

### 3.2. HepG2 Cells Transfected with LEG1 Genes

**Figure 1:**
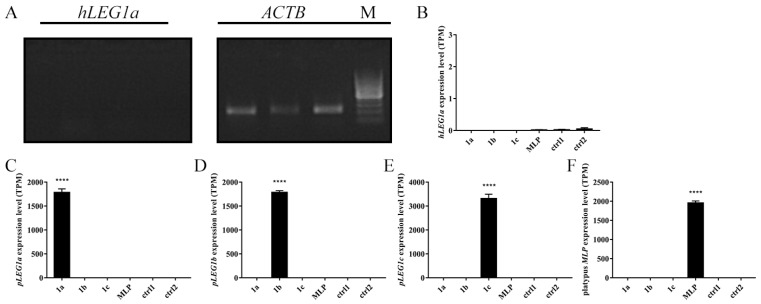
Detection of liver-enriched gene 1 (*LEG1*) gene expression in HepG2 cells. (**A**). Endogenous *hLEG1a* expression was not detected in HepG2 cells using RT-PCR. Beta-actin (*ACTB*) was used as an internal control. (**B**–**F**). The expression levels (transcripts per million, TPM) of *hLEG1a* (**B**), *pLEG1a* (**C**), *pLEG1b* (**D**), *pLEG1c* (**E**), and platypus *MLP* (**F**) were calculated in each cell transfection group by RNA-seq. Data are the means ± standard errors of the mean (SEMs) with **** indicating a *p*-value < 0.0001.

**Figure 2:**
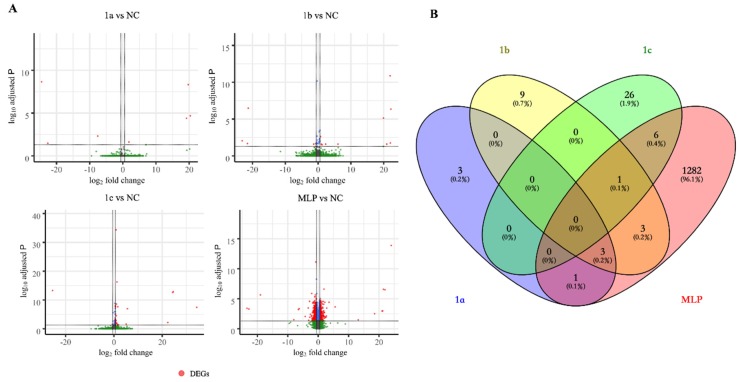
Comprehensive analysis of the RNA-seq results. (**A**) Volcano plots showing the differentially expressed genes (DEGs) identified from each *LEG1* transgenic group versus their respective controls. The DEGs, indicated by red dots, were identified according to an adjusted *p*-value < 0.05 and |log_2_ fold change| > 0.5849. (**B**) The Venn diagram of the DEGs shows that only a few genes were shared between groups.

**Figure 3:**
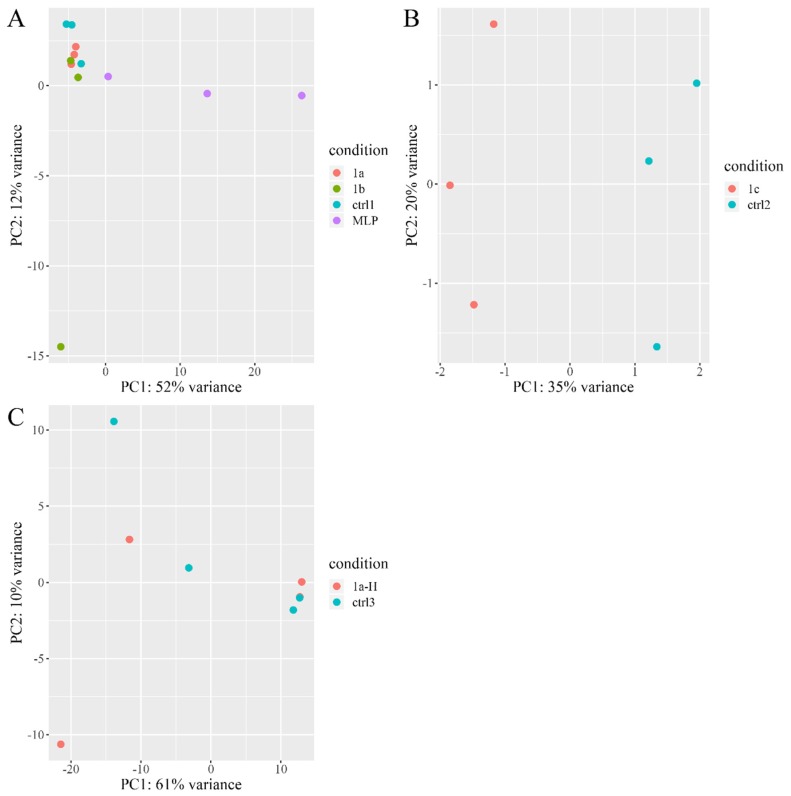
Scatter plot presenting the distribution of samples in the two-dimensional coordinate system determined by the first (PC1) and the second (PC2) principal components, which were calculated using the expression values normalized by the *vst* function implemented in DESeq2 R package. (**A**) PCA result of *pLEG1a* (1a), *pLEG1b* (1b), platypus *MLP* (MLP), and ctrl1. (**B**) PCA results of *pLEG1c* (1c) and ctrl2. (**C**) PCA result of HEK293T cells overexpressing *pLEG1a* (1a-H) or empty control (ctrl3).

As a human liver cancer cell line, the HepG2 cell line was used in our experiment to elucidate the function of “liver-enriched genes”. The lack of endogenous expression of *hLEG1a* shown by RT-PCR (Figure 1A) precluded the application of a loss-of-function study. Alternatively, a gain-of-function study by the overexpression of pig *LEG1s* and platypus *MLP* was performed. Two batches of experiments were conducted in series. In the first batch, the HepG2 cells were transfected with *pLEG1a*, *pLEG1b*, and platypus *MLP* expression plasmids and an empty vector (designated hereafter as the 1a, 1b, MLP, and ctrl1 groups); the second batch consisted of experiments using the *pLEG1c* plasmid and the empty vector (1c and ctrl2 hereafter). Total RNA was extracted 48 h later and subjected to RNA-seq analysis, in which the DEGs were analyzed using 1a, 1b, and MLP compared with ctrl1 and 1c compared with ctrl2. By comparing the RNA-seq readouts, each exogenous gene was found to be highly and specifically expressed in transfected cells, while endogenous *hLEG1a* levels were barely detectable, confirming the RT-PCR results (Figure 1). qRT-PCR was then employed to validate the RNA-seq results, in which the exogenous gene expression levels were comparable in each group (Table S3). RNA-seq analysis showed that in 1a cells, there were only 7 DEGs, among which 4 genes were upregulated, and 3 were downregulated; in the 1b group, 16 DEGs were found, among which 10 were upregulated, and 6 were downregulated; in 1c cells, 33 DEGs were detected, among which 31 were upregulated, and 2 were downregulated; in MLP transfected cells, 1305 DEGs were discovered, among which 539 were upregulated, and 766 were downregulated (Figure 2A). No DEGs were shared by the four groups. In addition, only a few genes were shared between groups, indicating that the functions of the genes might differ (Figure 2B). Next, a heatmap of the transcription profiles was constructed using all of the DEGs identified (1335 DEGs) in combination with the RNA-seq results from our previous HEK293T study, which served as an outgroup (GSE134920) [9]. The comparison showed that platypus *MLP* triggered significantly different transcriptomic alterations compared to the other groups (Figure S2). PCA analysis indicated that 1a and 1b were grouped with ctrl1, while the 1c and MLP groups exhibited more differences from their respective controls (Figure 3).

### 3.3. Enrichment Analysis Using DAVID and MSigD

The DEGs identified by RNA-seq were subjected to GO and KEGG analysis using DAVID and MSigD. For the 1a group, no hits were found using either of the methods (Table 1, Tables S4 and S5). For the 1b group, GO terms related to transcriptional regulation were identified using DAVID (Table 1 and Table S4). DAVID analysis of the DEGs from the 1c group showed that cell differentiation, autophagy, glucose transport, protein stability, and unfolded protein terms were enriched (Table 1 and Table S4). MSigD only showed that the endoplasmic reticulum (ER) stress-related process was changed in the 1c group (Table S5). Thus, *pLEG1c* might primarily regulate improperly folded proteins in the ER. More than 1300 DEGs were identified in the MLP group, resulting in numerous potentially disturbed pathways or biological processes. The GO_BP terms identified mitochondrial-related pathways such as mitochondrial translation, protein transport, respiratory chain complex I assembly, and electron transport; transcription and translation modulation; gene silencing; cell cycle regulation; organ, tissue, and cell development and differentiation; and neurogenesis and cerebral cortex development. The KEGG terms were associated with many cancer-related diseases, including renal cell carcinoma, viral carcinogenesis, and prostate cancer, neural system disorders, including Huntington’s disease, Alzheimer’s disease, Parkinson’s disease, and non-alcoholic fatty liver disease (Table 1 and Tables S4 and S5). Thus, platypus *MLP* might have broad biological functions related to the neural system, mitochondria, and development.

### 3.4. GSEA

To complement our enrichment analysis, the genes identified in the RNA-seq experiments were subjected to GSEA despite the significance levels. An additional term in the 1a group was detected that participates in the negative regulation of translational initiation (Table 2). Hormone biosynthesis, synapse localization, and systemic lupus erythematosus terms were identified in the 1c group (Table 2). GSEA of platypus *MLP* showed terms such as detoxification, defense response to fungus, defense response to Gram-negative bacterium, antigen processing and presentation, and virion assembly, implying an anti-extracellular biotic stress function of *MLP* (Table 2 and Table S6).

## 4. Discussion

Our previous study suggested that mammalian *LEG1* genes could be divided into three classes, *LEG1a*, *LEG1b*, and *LEG1c* [9]. Tandem duplication occurred first in the common ancestor of mammals, giving rise to the *LEG1c* and *LEG1* branches. Then, another duplication event took place in the common ancestor of eutherian mammals. Therefore, three copies of *LEG1* can be found in some eutherian species, such as pig, while only two copies existed in proto- and metatherian species, such as the platypus. However, in the majority of eutherian animals, only the *LEG1a* gene has been preserved due to the pseudogenization of *LEG1b* and *LEG1c* (e.g., *hLEG1a* and *mLeg1a*). Therefore, the pig might be a better model for elucidating the functions of each *LEG1* clade, since it retains all *LEG1* copies [9]. Thus, *pLEG1a*, *pLEG1b*, and *pLEG1c* were subjected to subsequent studies.

Thus far, functional studies regarding *LEG1* have only been conducted on *zleg1* and platypus *MLP* [1,2,8,19]. In the former species, *zleg1* genes were suggested to play a role in liver development, since the knockdown of *zleg1* genes results in a small liver phenotype [1]. In the latter species, *MLP* shows antibacterial activity in milk [8]. No functional studies on eutherian *LEG1* have yet been published. Hence, to determine the potential roles of the genes, bioinformatics tools were employed first to predict the functions of the *pLEG1* genes. As prediction methods are all based on a priori knowledge, each LEG1 protein was predicted to exhibit nearly identical functions using each approach. This may be because the LEG1 proteins constitute a protein family containing no other proteins, and the different paralogs could not be discerned by in silico prediction methods. PANDA predicted that platypus *MLP* and pig *pLEG1c* are involved in digestive system/liver development, which may be based on the study of *zleg1*. However, in our study, we did not find any entries related to liver or digestive system development except for non-alcoholic fatty liver disease entry in *MLP*-transfected cells (Table S4). In contrast, the I-TASSER method predicted that LEG1 proteins play roles in the mitochondria, respiration, heme binding, and neural system, as related terms were identified in our experiment (Tables S4–S6).

According to previous transcription profiling experiments, *hLEG1a*, *mLeg1a*, and *pLEG1a* could only be detected in the salivary glands, while *pLEG1b* and *pLEG1c* were not detectable in the examined tissues, in contrast to the ubiquitous expression of platypus *MLP* [8,9,20,21]. Thus, we proposed in our previous paper that after the duplication events, the mammalian *LEG1* genes underwent subfunctionalization, leading to different functions of each of the *LEG1* copies [9,22,23]. Therefore, we initially decided to study each clade of *LEG1* genes in the liver or salivary gland epithelial cells. However, no authenticated immortalized salivary gland epithelial cell lines exist at present, as previously established cell lines have been reported to be contaminated by other cells or are not well characterized or widely used [24,25,26,27]. Therefore, only the HepG2 liver cell line was employed in our current study. As demonstrated by RT-PCR, no endogenous *hLEG1a* expression was detected in the cell line, and the gain-of-function approach by the overexpression of *pLEG1* genes and platypus *MLP* in the cells was utilized.

By the overexpression of *LEG1* genes in HepG2 cells, gene functions could be predicted based on the altered transcriptomes [28]. Our RNA-seq analysis confirmed the highly specific expression of each *LEG1* gene in the cells 48 hours after transfection (Figure 1 and Table S3). Additionally, the expression levels of each *LEG1* gene detected by qRT-PCR were comparable, precluding the effect of different expressions on the altered transcriptomes (Table S3). Then, the DEGs were first used in searches against DAVID [12,13] and MSigD [14] databases by the simple Fisher’s exact test for overrepresented biological processes archived in the GO [15] and KEGG [16] databases. Moreover, the RNA-seq experiment revealed either a few DEGs or numerous DEGs, which are more suitable for GSEA [14] irrespective of the number of DEGs. Thus, GSEA was employed to provide additional information regarding the functions of the *LEG1* genes. In order to provide a more comprehensive prediction of the *pLEG1a* functions, loose parameters (the *p*-values rather than the adjusted *p*-values), were utilized to identify the DEGs in HEK293T cells transfected with *pLEG1a* in our previous work, in spite of more false-positive hits [9]. To reduce the false-positive prediction, our current work used the stringent parameters. In *pLEG1a*- and *pLEG1b*-overexpressing cells, only 7 and 16 DEGs were identified, which showed potential roles in translation or transcriptional regulation, respectively, using different enrichment approaches. However, due to the relatively small number of DEGs and altered pathways/biological processes identified, in combination with the PCA results showing the clustering of the 1a and 1b groups with the ctrl1 group, *pLEG1a* and *pLEG1b* may not be functional in liver cells, confirming a subfunctionalization model from our previous study [9]. Regarding the *pLEG1c* gene, the functional predictions using GO and KEGG showed that it might play a major role in ER stress and misfolded protein regulation, with additional roles in autophagy, cell differentiation, and glucose transport. However, these functions could be dispensable since *pLEG1c* was not expressed in the tissues tested [9]. As platypus *MLP* could be detected in several tissues, including the liver [8], the examination of platypus *MLP* revealed numerous disturbed GO and KEGG terms, suggesting its role in organ/tissue/cell development, the neural system, cell cycle regulation, and mitochondria-related pathways, consistent with the prediction results of I-TASSER. Platypus *MLP* and *pLEG1c* are orthologs; thus, some potential biological roles were shared by the two genes, such as autophagy and neuron-related processes. One interesting role of platypus *MLP* is its antibacterial activity, and in our analysis some immune-related pathways and viral, fungal, and bacterial-related pathways were also identified (Table 2). In the platypus MLP experiment, the protein showed antibacterial activity against Gram-positive bacteria, with no effect on Gram-negative microbes, which is contrary to the anti-Gram-negative bacteria GO term (Table 2) [8]. Therefore, further experimental validation is needed. In the current study, we did not find that *pLEG1c* was associated with antibacterial activity, nor did the *pLEG1s* or platypus *MLP* show any functional roles in liver development compared to the orthologous zebrafish *leg1s*. It has been reported that *LEG1* genes exhibit differential expression patterns and functional differences. In addition, strong purifying selection across vertebrate *LEG1* genes has also been noticed [9]. Similar results have been presented in other genes as well [29,30], which suggests subfunctionalization events after gene duplications [22,23]. Therefore, each of the *LEG1* duplicates retained a subset of ancestral *LEG1* gene function.

## 5. Conclusions

Using RNA-seq-based prediction methods, it was found that *pLEG1* genes, as representatives of mammalian *LEG1* genes, may exert different functions in liver cells, confirming a subfunctionalization event during evolution.

## Figures and Tables

**Table 1:** Significantly enriched Gene Ontology (GO) and Kyoto Encyclopedia of Genes and Genomes (KEGG) terms/pathways identified by DAVID.

Group	Category	Term/Pathway	*p*-Value
1b	GO_BP	Negative regulation of transcription from RNA polymerase II promoter	0.01
	GO_BP	Regulation of transcription from RNA polymerase II promoter	0.03
	GO_MF	Transcription factor activity, sequence-specific DNA binding	0.027
1c	GO_BP	Regulation of protein complex stability	0.009
	GO_BP	autophagy	0.018
	GO_BP	Cell differentiation	0.034
	GO_BP	Positive regulation of glucose import	0.045
	GO_BP	Protein secretion	0.048
	GO_CC	Endoplasmic reticulum	0.001
	GO_CC	Endoplasmic reticulum chaperone complex	0.017
	GO_CC	Endoplasmic reticulum lumen	0.035
	GO_CC	Smooth endoplasmic reticulum	0.039
	GO_MF	Protein binding	9 × 10^−4^
	GO_MF	Unfolded protein binding	0.01
	GO_MF	Growth factor activity	0.02
	KEGG	Protein processing in endoplasmic reticulum	2.9 × 10^−4^
MLP	GO_BP	translation	1.6 × 10^−13^
	GO_BP	Mitochondrial translational termination	4.2 × 10^−6^
	GO_BP	Protein import into mitochondrial inner membrane	0.002
	GO_BP	Mitochondrial respiratory chain complex I assembly	0.005
	GO_BP	Cell cycle arrest	0.014
	GO_BP	Cerebral cortex development	0.021
	KEGG	Ribosome	3.3 × 10^−15^
	KEGG	Huntington’s disease	2.6 × 10^−4^
	KEGG	Alzheimer’s disease	0.001

**Table 2:** Significantly enriched GO and KEGG terms/pathways identified by gene set enrichment analysis (GSEA).

Group	Up/Down Regulated DEGs	Category	Term/Pathway
1a	Up	GO	Negative regulation of translational initiation
1c	Up	GO	Regulation of response to endoplasmic reticulum stress
		GO	Chaperone mediated protein complex assembly
		GO	Smooth endoplasmic reticulum
		GO	Cellular response to topologically incorrect protein
		GO	Protein localization to synapse
		GO	Protein folding in the endoplasmic reticulum
		GO	Unfolded protein binding
		GO	IRE1 mediated unfolded protein response
		KEGG	Steroid hormone biosynthesis
	Down	KEGG	Systemic lupus erythematosus
MLP	Up	GO	Detoxification
		GO	Defense response to fungus
		GO	Defense response to Gram-negative bacterium
		GO	Antigen processing and presentation
		GO	Virion assembly
	Down	GO	Low-density lipoprotein particle binding
		KEGG	Adherens junction

## Supplementary Materials

The following are available online at https://www.mdpi.com/2073-4425/11/4/412/s1, Figure S1. Schematic representation of the four *LEG1* containing plasmids. Figure S2. Heatmap showing the expression profiles of 1335 DEGs in each *LEG1* transgenic group versus the respective controls. Hierarchical clustering was applied to the samples (columns) and DEGs (rows). The HEK293T cells with *pLEG1a* expression (1a-H1, -H2, -H3, and -H4) and without *pLEG1a* expression (ctrl 3-1, -2, -3, and -4) were used as outgroups. Table S1. PANDA prediction of the functions of LEG1s. Table S2. TASSER prediction of the functions of LEG1s. Table S3. qRT-PCR validation of *LEG1* gene expression in each group. Table S4. Complete list of significantly enriched GO and KEGG terms/pathways identified by DAVID. Table S5. Complete list of significantly enriched GO terms identified by MSigD. Table S6. Complete list of significantly enriched GO and KEGG terms/pathways identified by GSEA.

## Abbreviations

LEG1	liver-enriched gene 1
MLP	monotreme lactation protein
DUF781	domain of unknown function 781
GO	Gene Ontology
KEGG	Kyoto Encyclopedia of Genes and Genomes
DEG	differentially expressed gene
BP	biological process
CC	cellular component
MF	molecular function
PCA	principal component analysis
DAVID	Database for Annotation Visualization and Integrated Discovery tool
MSigD	Molecular Signature Database
GSEA	Gene set enrichment analysis
TPM	transcripts per million
NES	Normalized enrichment score
ER	Endoplasmic reticulum
