# Supplementary material for: Transcriptomic Prediction of Pig Liver-Enriched Gene 1 Functions in a Liver Cell Line
Source: Genes (Basel). 2020 Apr 10;11(4):412. doi: 10.3390/genes11040412 (PMC7230230; doi:10.3390/genes11040412)

**Figure S1.** Schematic representation of the four *LEG1* containing plasmids.


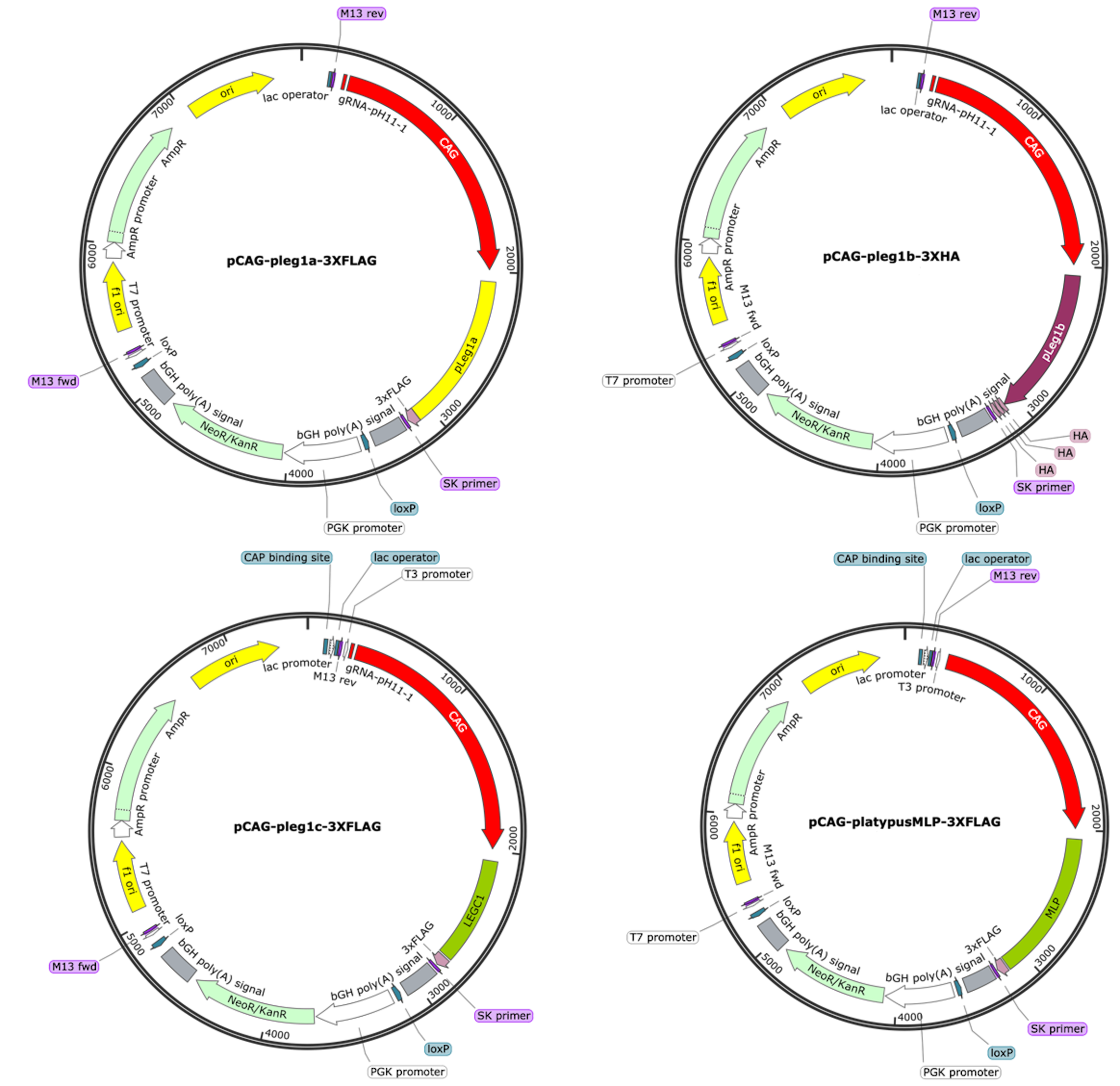


**Figure S2.** Western blot validation of the translation of transgenic *LEG1* genes in each group. Ctrl: 1:1 mixture of ctrl1 and ctrl2; pleg1a, pleg1b, MLP, pleg1c: *pLEG1a*, *pLEG1b*, *pLEG1c*, platypus *MLP* overexpressed groups, respectively.

**
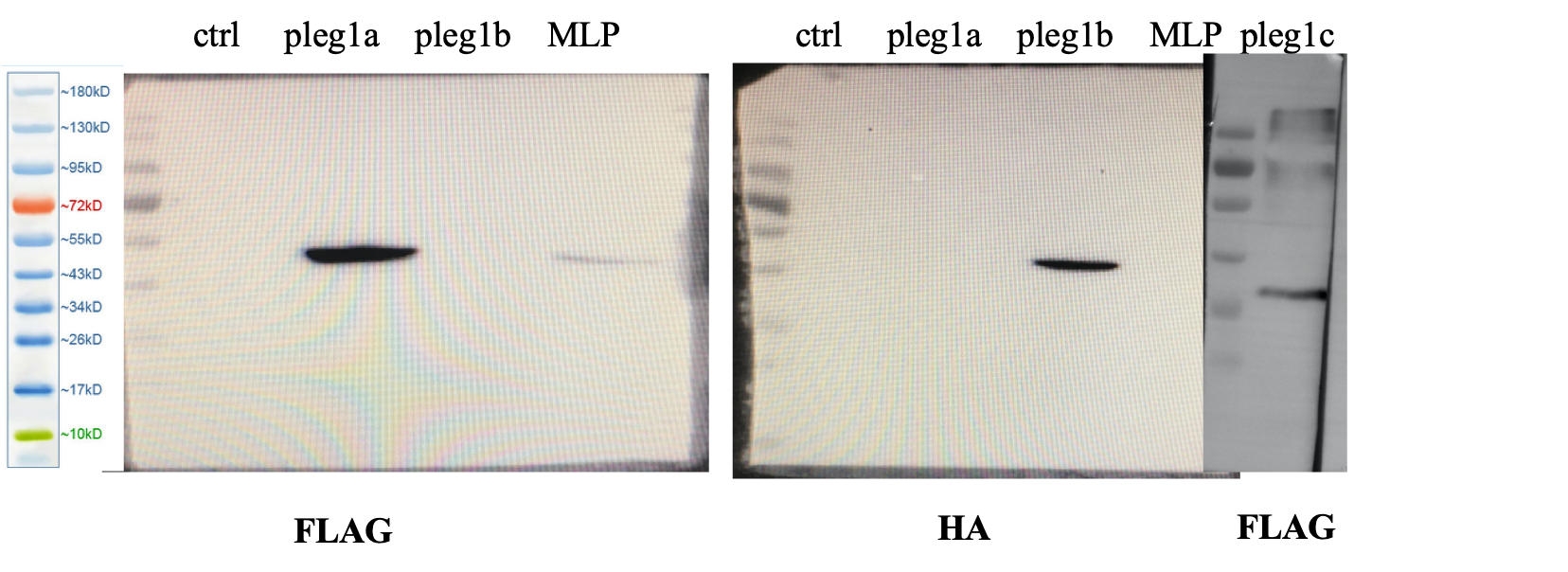
**

**Figure S3.** Heatmap showing the expression profiles of 1335 DEGs in each *LEG1* transgenic group versus the respective controls. Hierarchical clustering was applied to the samples (columns) and DEGs (rows). The HEK293T cells with *pLEG1a* expression (1a-H1, -H2, -H3, and -H4) and without *pLEG1a* expression (ctrl 3-1, -2, -3, and -4) were used as outgroups.


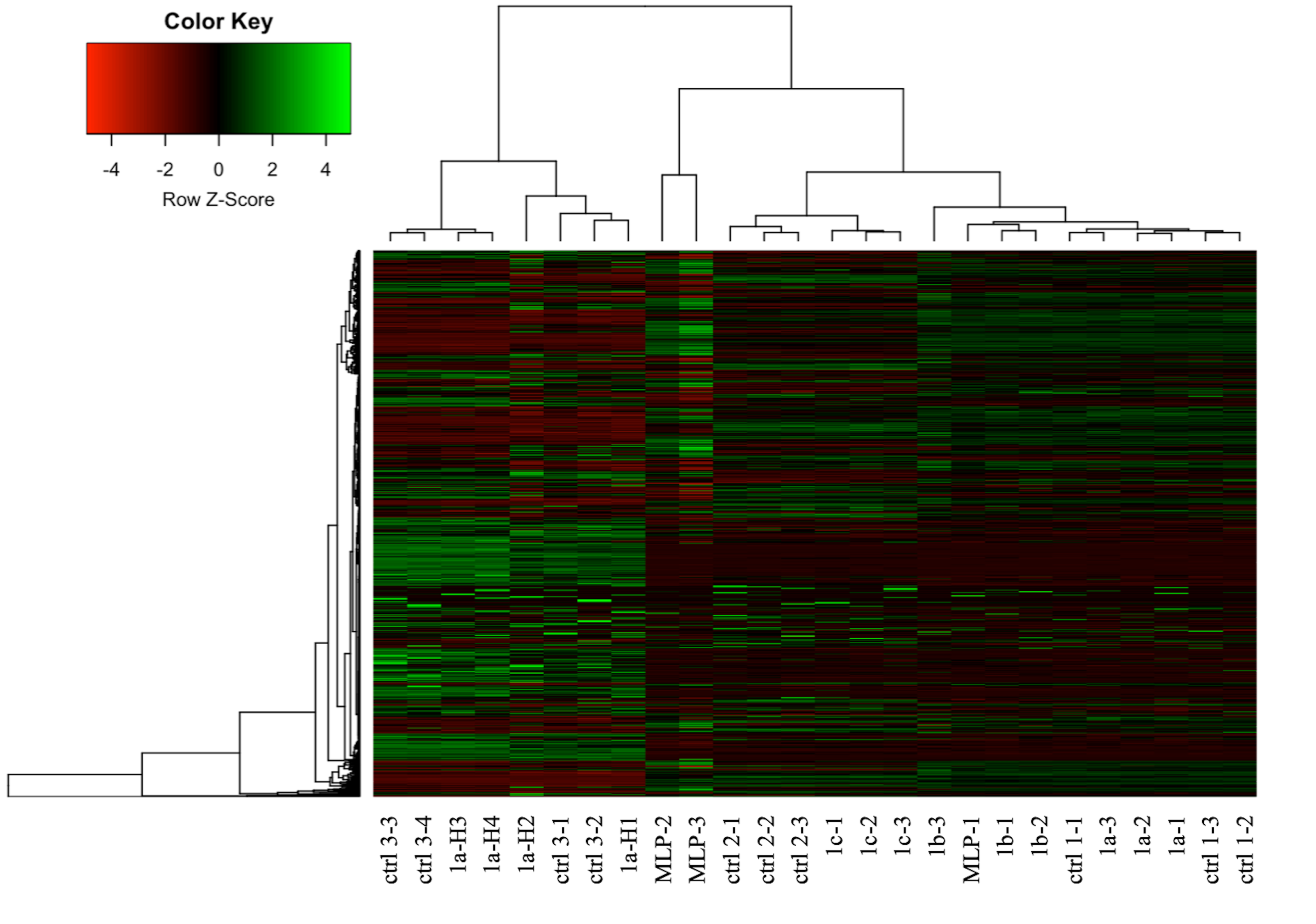

Supplement: Supplementary file 1 [file genes-11-00412-s001.zip › Supplementary Figure.docx]
